# Controllable growth of vertically aligned graphene on C-face SiC

**DOI:** 10.1038/srep34814

**Published:** 2016-10-06

**Authors:** Yu Liu, Lianlian Chen, Donovan Hilliard, Qing-song Huang, Fang Liu, Mao Wang, Roman Böttger, René Hübner, Alpha T. N’Diaye, Elke Arenholz, Viton Heera, Wolfgang Skorupa, Shengqiang Zhou

**Affiliations:** 1 Helmholtz-Zentrum Dresden-Rossendorf, Institute of Ion Beam Physics and Materials Research, 01328 Dresden, Germany; 2 Department of Basic Science, Beijing Information Science and Technology University, Beijing 100192, China; 3 School of Physics, Dublin Institute of Technology, Dublin, Ireland; 4 School of Chemical Engineering, Sichuan University, Chengdu 610065, China; 5 Technische Universität Dresden, 01062 Dresden, Germany; 6 Advanced Light Source, Lawrence Berkeley National Laboratory, Berkeley, California 94720, USA

## Abstract

We investigated how to control the growth of vertically aligned graphene on C-face SiC by varying the processing conditions. It is found that, the growth rate scales with the annealing temperature and the graphene height is proportional to the annealing time. Temperature gradient and crystalline quality of the SiC substrates influence their vaporization. The partial vapor pressure is crucial as it can interfere with further vaporization. A growth mechanism is proposed in terms of physical vapor transport. The monolayer character of vertically aligned graphene is verified by Raman and X-ray absorption spectroscopy. With the processed samples, d^0^ magnetism is realized and negative magnetoresistance is observed after Cu implantation. We also prove that multiple carriers exist in vertically aligned graphene.

Graphene is a 2D carbon allotrope with honeycomb monolayers. This kind of unique structure exhibits amazing electronic, photo-electric, magnetic, mechanical, and chemical properties^1^, which makes graphene one of the most interesting research areas since its discovery^2^. So far, by composite^3^,^4^, doping^5^ or structural control^6^,^7^,^8^, graphene has impacted various application fields, such as energy storage^5^,^6^,^7^,^8^,^9^, catalysis^3^, and biosensors^4^, and nonvolatile device^10^.

Vertically aligned graphene^11^ is a configuration of nearly free-standing graphene nanostructures as its internal graphene nanostrips are self-supporting, thus, limiting the influence of substrates. Its large surface-to-volume ratio and numerous reactive edges along with the extraordinary intrinsic properties of graphene bring potential applications in field emission sources^12^,^13^, electrodes for lithium-ion batteries^14^, thermal interfacial materials^15^ and supercapacitors^16^,^17^,^18^,^19^,^20^,^21^. Currently, vertically aligned graphene can be prepared by several methods. Chemical vapor deposition can grow vertically aligned graphene on different substrates, so it can be used to decorate the surface to improve the performance of materials^14^,^16^,^17^,^19^,^20^,^21^,^22^; for a recent review, see the relevant part in ref. 23. Reprocessing obtained graphene is a practical way to produce massive samples rapidly^15^,^18^. Another sophisticated method fabricates vertical graphene arrays using chromonic liquid crystal precursors followed by carbonization^24^. Thermal decomposition of C-face or non-polar face (10-10)/(11-20) SiC can produce vertically aligned graphene sheets. As this method achieves high purity and alignments of the samples, such samples are well-suited to study the intrinsic properties of graphene^11^,^25^. With vertically aligned graphene on SiC, marvelous cold cathode field emission performance has been realized^12^,^13^. Anharmonic phonon effects have been observed in the temperature range between 79 and 773 K using Raman spectroscopy^26^. Intrinsic nonlinear diamagnetism has been verified along with other magnetic properties^27^,^28^. Moreover, anisotropic quantum transport has been revealed in vertically aligned graphene on SiC as well as on Si substrates^29^,^30^.

Although the properties of vertical graphene have been studied well, its growth on SiC is hard to control and its growth mechanism is not clear, which will hinder its further development and application. To promote the potential application, it is necessary to determine how process parameters (such as temperature and pressure) affect the graphene growth. Here, we investigated the growth of vertically aligned monolayer-like graphene on C-face SiC. The influence of temperature, annealing time, temperature gradient, pressure, and crystalline quality of SiC substrates was studied. Based on the growth, the magnetism and transport properties in as-grown and Cu-implanted vertical graphene were explored.

## Results

### Temperature and annealing time

**Figure 1:**
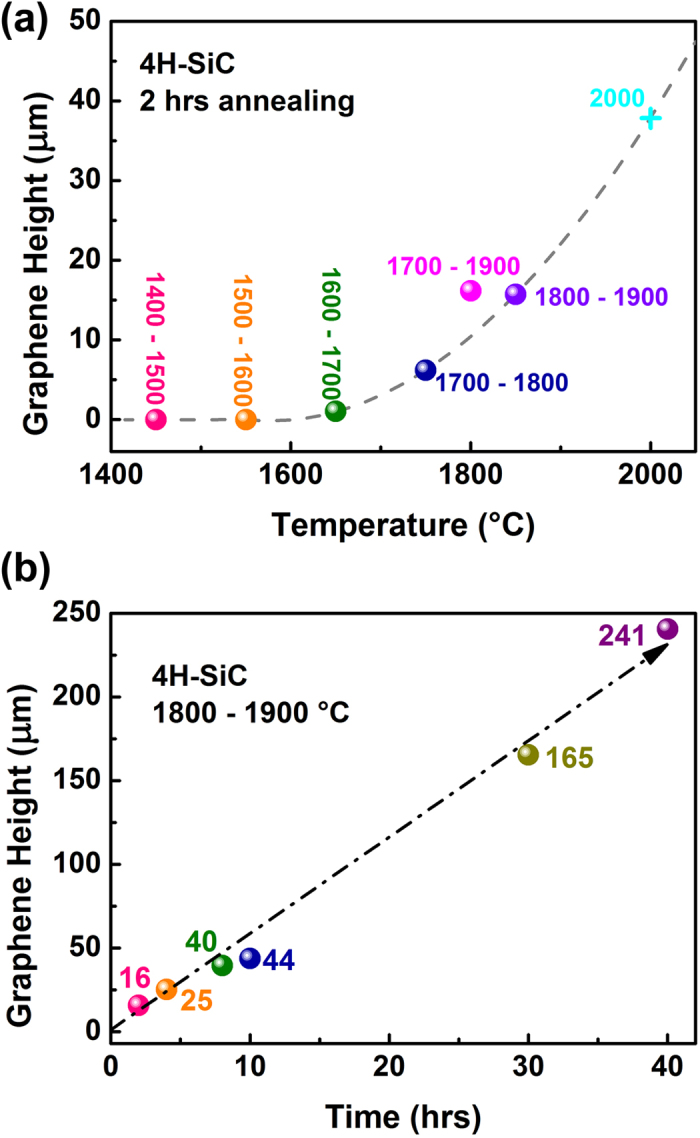
The height of vertically aligned graphene vs. annealing temperature and time: (**a**) Graphene height as a function of process temperature. The balls representing the results are located at the average temperature during annealing. Numbers indicate the actual temperatures in the processes. The grey dashed line is a guide to eyes. The cross indicates the expected graphene height at an annealing temperature of 2000 °C. (**b**) Graphene height as a function of annealing time. The arrow is a guide to eyes. The numbers correspond to the height of each sample.

To confirm the temperature threshold required to grow the vertically aligned graphene, the substrates were annealed at different temperatures for 2 hours between 1400 °C and 1900 °C. No graphene was obtained using temperatures of 1400 °C to 1600 °C. Vertically aligned graphene begins to grow when the temperature reaches 1700 °C. With further increasing temperature, graphene growth speeds up. We find that the growth rate increases with temperature. The height of the obtained graphene in different samples is listed in Fig. 1(a). According to this trend, a fully carbonized samples (usually around 330 μm) is expected to be prepared in 20 hours at a temperature of 2000 °C. We varied the initial annealing temperature to allow for a sharper temperature increase, i.e.: 1700 °C–1900 °C compared to 1800 °C–1900 °C. The results show that the graphene height is mainly determined by the final temperature.

Usually, epitaxial planar graphene is compact and it will prevent the vapor release, so planar graphene cannot grow too thick. However, the situation for vertically aligned graphene is unclear. Can tunnels inside the vertical graphene increase or decrease the sublimation rate? The vertically aligned graphene was prepared with annealing times varying up to 40 hours, as shown in Fig. 1(b). Graphene samples with a height of up to 241 μm were obtained. The result demonstrates that the growth rate is nearly constant, i.e. it is mainly determined by the temperature. Therefore, the grown vertical graphene does not promote or prevent the vaporization. It has almost no influence to the subsequent growth.

### Longitudinal temperature gradient

**Figure 2:**
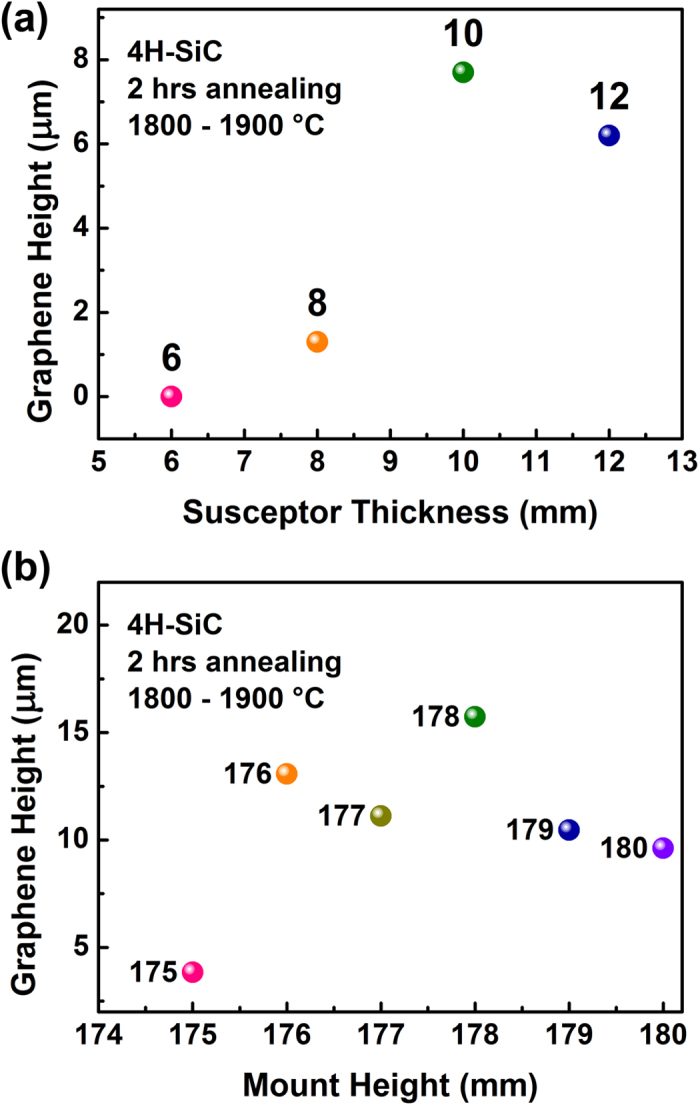
The graphene height vs. the susceptor thickness (**a**) and mount height (**b**). The numbers represent the susceptor thickness or mount height. The mount height is fixed at 175 mm when the susceptor thickness varies; the susceptor thickness is 6 mm when the mount height changes. The substrates are located in the middle of the susceptors when graphene grew with different susceptor thicknesses, while they are at the edge when different mount heights are applied. Therefore, the former graphene heights are generally smaller than the later ones.

**Figure 3:**
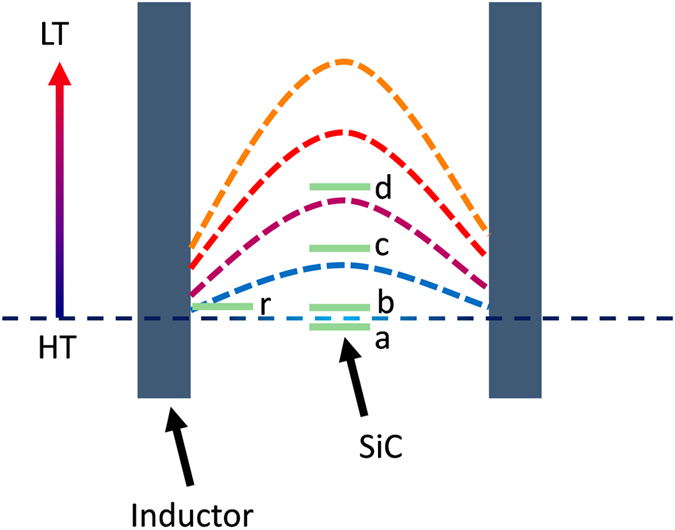
Schematic to illustrate the relative positions of the substrates in the temperature gradient. The labels a - d correspond to the four cases mentioned in the results on the influence of the longitudinal temperature gradient. The label r represents the case when the substrate is located at the edge of the susceptor. The inductor is the outer lateral surface of the susceptor and its lid in this growth system. This schematic should not be considered to represent the realistic positions of substrates and the realistic temperature gradient inside the space surrounded by the susceptor and its lid.

The crystal growth is sensitive to the temperature distribution in the crucible^31^. The growth of vertically aligned graphene could also be affected by the temperature gradient inside the space surrounded by the susceptor and its lid. First, we checked the influence of the longitudinal temperature gradient. The bottom thickness of the susceptor and the height of the graphite tubular mount (Hereinafter, referred to as the susceptor thickness and the mount height.) determine the vertical position of substrates, so they more or less determine the temperature gradient. The initial susceptor thickness and mount height are 6 mm and 175 mm, respectively (Figures S1 and S2, Supplementary Information). With this setup, no vertical graphene was obtained, when the C-side was faced up. Only planar graphene layers grew on the Si-face. In contrast, if the C-side is put facing down, vertically aligned graphene can be obtained on the C-face but the graphene height is rather limited. This difference does not happen in the planar graphene fabrication. It can only be understood by the temperature gradient as the temperature in the place of the substrates should be unchanging. This indicates that the temperature gradient plays an important role in the growth of vertically aligned graphene. To further understand this mechanism, susceptors with different bottom thicknesses as well as mounts with different heights were employed to grow graphene. The results in Fig. 2 suggest that the maximum growth rate can be achieved by varying the vertical position of the substrates. The influence of the longitudinal temperature gradient can be understood like this (see schematic in Fig. 3):

- When the C-side is faced up with the initial setup (6 mm of susceptor thickness and 175 mm of mount height), the C-side faces towards the temperature maximum and the vapor cannot escape as it usually moves from high to low temperature. So no graphene can grow during annealing.
- When the C-side is faced down with the initial setup, the substrates cross the position of temperature maximum, the vapor can escape easily and then vertically aligned graphene can be obtained.
- When the susceptor thickness or the mount height increases, the substrates move away from the temperature maximum, the temperature gradient increases and graphene can grow faster.
- When the susceptor thickness or the mount height continuously increases, the substrates move further away, the temperature drops and then the growing rate decreases, considering that the difference is dramatically increased between the sample temperature and the measured temperature.

Therefore, altering the position of the substrates is an important means to control the growth of vertically aligned graphene.

### Radial temperature gradient

The substrates were loaded both in the middle and at the edge of the susceptor to grow graphene simultaneously for 8 hours. The height of the graphene grown in the middle is 22 μm compared with 58 μm at the edge. As the heat is generated only on the outer lateral surface of the susceptor and the lid, the edge will have both higher temperature and higher temperature gradient than the inner part, which can accelerate graphene growth. However, the direction of temperature gradient is not perpendicular to the substrate surface, which may result in a height inhomogeneity in lateral direction. It is also noticed that, the substrates are located in the middle of the susceptors when graphene grew with different susceptor thicknesses, while they are at the edge of the susceptors when different mount heights are applied. Therefore, the former graphene heights are smaller than the latter ones because of the relatively low temperature and relatively gradual temperature gradient (See Fig. 2).

### Pressure

The residual pressure in the chamber also affects the growth of vertically aligned graphene. For this investigation, we used a mechanic pump instead of the turbo molecular pump. The pressure maintained at about 9 mbar. With the susceptor thickness of 6 mm and the mount height of 178 mm, the graphene height reduces from 15.7 μm to 9.4 μm. It can be understood that when the pressure increases, the vapor partial pressure around the substrates rises and the convection decreases the temperature gradient, both of which will suppress the sublimation. If several pieces of substrates are annealed at one time, the height of the obtained graphene will also decrease. For instance, the height was 28 μm after annealing 8 pieces for 8 hours, compared with 40 μm for annealing only 1 piece, as the total Si-evaporation from 8 pieces raises the Si-partial pressure higher than that of 1 piece and it will suppress the sublimation on each single sample. This result reveals the evacuation of the vapor created from the surface of substrates is critical to the growth of vertically aligned graphene.

### Substrates

It is known that the arrangements of vertical graphene arrays are different when grown on substrates with different crystallographic orientation^11^. It is further found that the growth on 6H-SiC is much faster than that on 4H-SiC (29 μm vs. 16 μm). The growth rate of graphene on substrates of production grade is also larger than that of dummy grade (Average height of 40 μm for 6 pieces annealing for 8 hours vs. 40 μm for 1 piece if the influence of the pressure is under consideration mentioned above). Usually, 6H-SiC substrates have better crystallinity than 4H-SiC ones due to the higher temperature of the growth surface^32^. The general crystal quality of wafers with production grade is also better than that of dummy grade (Figure S3, Supplementary Information). Therefore, it suggests that vertically aligned graphene tends to grow quickly on substrates with good quality. It is speculated that good crystalline quality can promote vaporization.

## Discussion

### Growth mechanism

**Figure 4:**
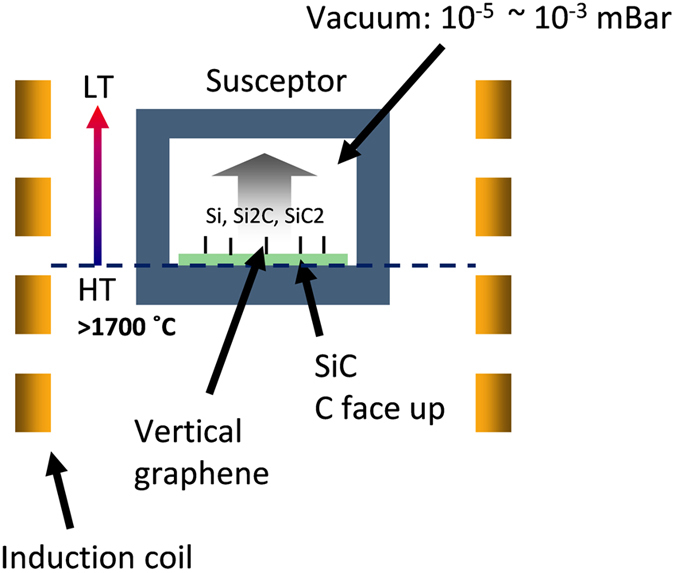
Schematic to illustrate the mechanism for the growth of vertically aligned graphene.

According to the results above, the growth of vertically aligned graphene can be understood in terms of SiC single crystal growth using the physical vapor transport method with a single wall crucible^33^. When it is heated at a temperature of more than 1080 °C^34^,^35^,^36^, SiC will sublimate without melting. (Temperatures around 1000 °C are important in SiC, as both sublimation and crystallization can occur depending on the pressure^37^,^38^). If the vapor (mainly SiC_2_, Si_2_C, Si and C) can be transported rapidly with the proper temperature gradient discussed above (Some may deposit on the inner surface of the lid.), the sublimation will continue. As the saturation vapor pressure of Si is higher than that of C at the same temperature, carbon will be left and will reconstruct. The reconstruction of C atoms resulting in vertically aligned graphene is more complicated. Considering the difference between the temperature, the temperature gradient, and the pressure utilized to prepare planar graphene and those of vertically aligned graphene, the vaporization rate could be the key factor in determining the structure after reconstruction. When the vaporization is high enough, the initial C atom sheets will vertically stand due to the strong gas flow. So graphite or planar graphene cannot form but only the low density of vertical graphene makes the crystallization possible. The guide of the high speed gas flow, the annealing under a high temperature, and the etching of Si atoms will further improve the standing thin film into vertically aligned graphene. The schematic is shown in Fig. 4. Therefore, now it is understood why the graphene height is rather limited when we annealed the substrates with the Si-side faced up using the initial susceptor thickness of 6 mm and mount height of 175 mm mentioned in the results on the influence of the longitudinal temperature gradient: the generated vapor cannot be evacuated immediately when it has to go through the space between substrates and the susceptor.

### Monolayer behavior

**Figure 5:**
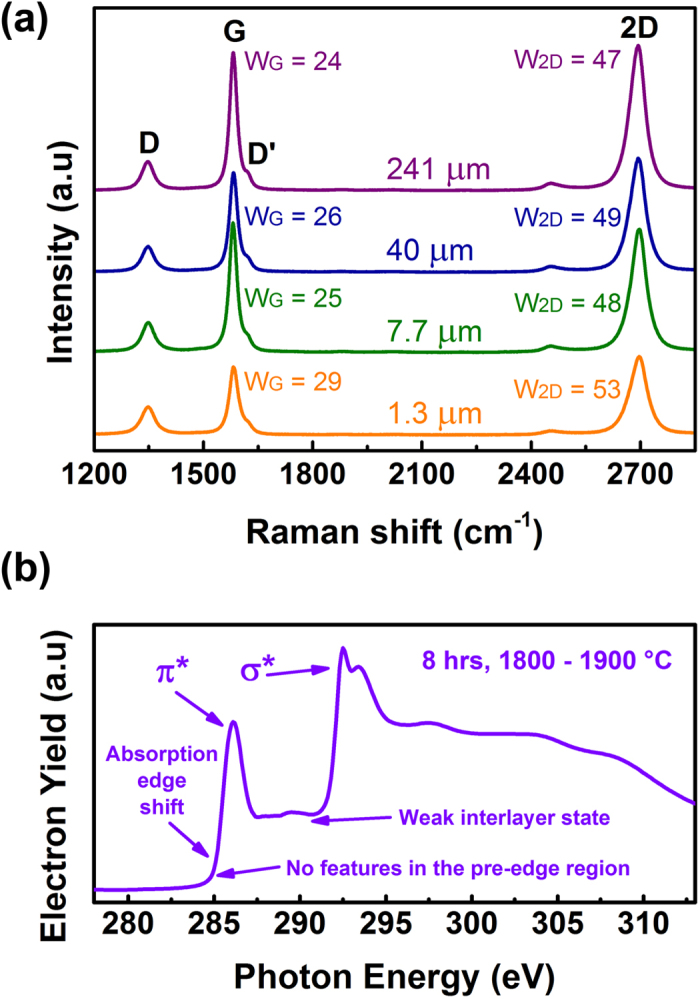
(**a**) Raman spectra for four vertically aligned graphene samples (Table S1, Supplementary Information). The monolayer behavior is verified by the symmetric Lorentzian 2D peaks. (**b**) The typical XAS spectrum at the C K-edge. The spectrum agrees well with the calculated spectrum of a graphene monolayer.

Raman spectra from all samples have four peaks G, D, D’ and 2D, which are the fingerprints of graphene, as shown in Fig. 5(a). The emergence of the D and D’ peaks suggests that the top caps may be very thin so the edge of the vertical graphene is almost exposed on as-grown samples^11^. Full width at half maximum (FWHM) of G and 2D peaks (W_G_ and W_2D_) from 24 to 29 cm^−1^ and from 47 to 53 cm^−1^ shows the similarly high quality of all samples. The 2D peaks are symmetric and well match a Lorentzian line, so the interaction between the adjacent layers is weak and graphene layers exhibit a monolayer behavior. It is heeded that the difference between measured samples is not only limited to their graphene height. They were prepared under different conditions: the 241 μm sample has grown for 40 hours; the 40 μm sample used 1700–1800 °C for 10 hours; and the Si-face of the 1.3 μm sample was up during the process (More details in Table S1, Supplementary Information). Therefore, altering the configuration of the growth has little effect on the quality of graphene samples. The results prove the robustness of the growth method and the growth system.

A typical XAS spectrum of a sample annealed for 8 hours is shown in Fig. 5(b). The C K-edge is located at 285 eV without any features in the pre-edge region. Two main peaks at 286 and 293 eV are assigned to π* and σ* resonance, respectively. The signal corresponding to interlayer states is weak. The XAS spectrum agrees well with the theoretical prediction of graphene monolayers^39^. It is consistent with the Raman result.

### Magnetic and electronic properties of as-grown and Cu-implanted vertically aligned graphene

**Figure 6:**
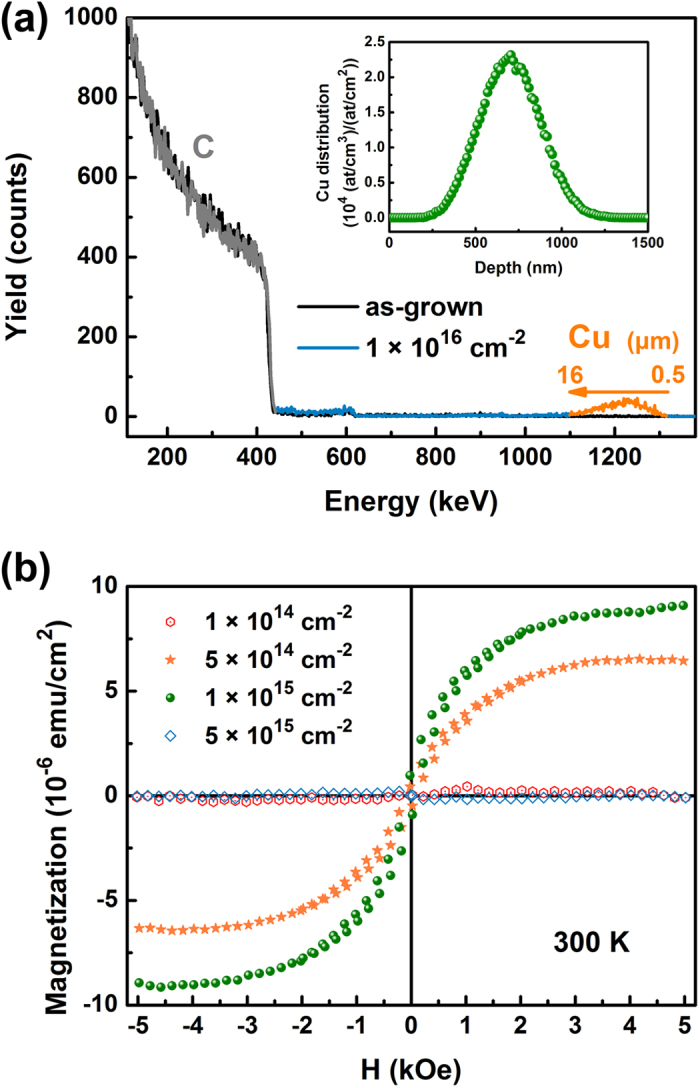
(**a**) Typical random RBS spectra from the as-grown and 1 × 10^16^ cm^−2^ sample. Inset: Cu distribution predicted using TRIM. (b) The magnetization as a function of magnetic field in Cu-implanted vertical graphene without the diamagnetic background.

As graphene is a one atom layer, the traditional doping methods are not effective. But it is possible to dope vertically aligned graphene, for instance, with ion implantation. We tried to induce d^0^ magnetism in vertical graphene by Cu implantation. The samples have been annealed for four hours before implantation and thus have the graphene height of 22 μm. The RBS spectra before and after implantation are shown in Fig. 6(a). There is no difference between the two spectra except the appearance of the Cu peak. The Cu distribution is determined to be in the range from 0.5 to 16 μm in contrast to the predicted range of 530 to 870 nm using TRIM (transport of ions in matter) simulation, which may be caused by the channeling effect due to the well alignment of vertical graphene. It is also noted that the density of vertical graphene at the surface is slightly lower than that deeper in the bulk.

**Figure 7:**
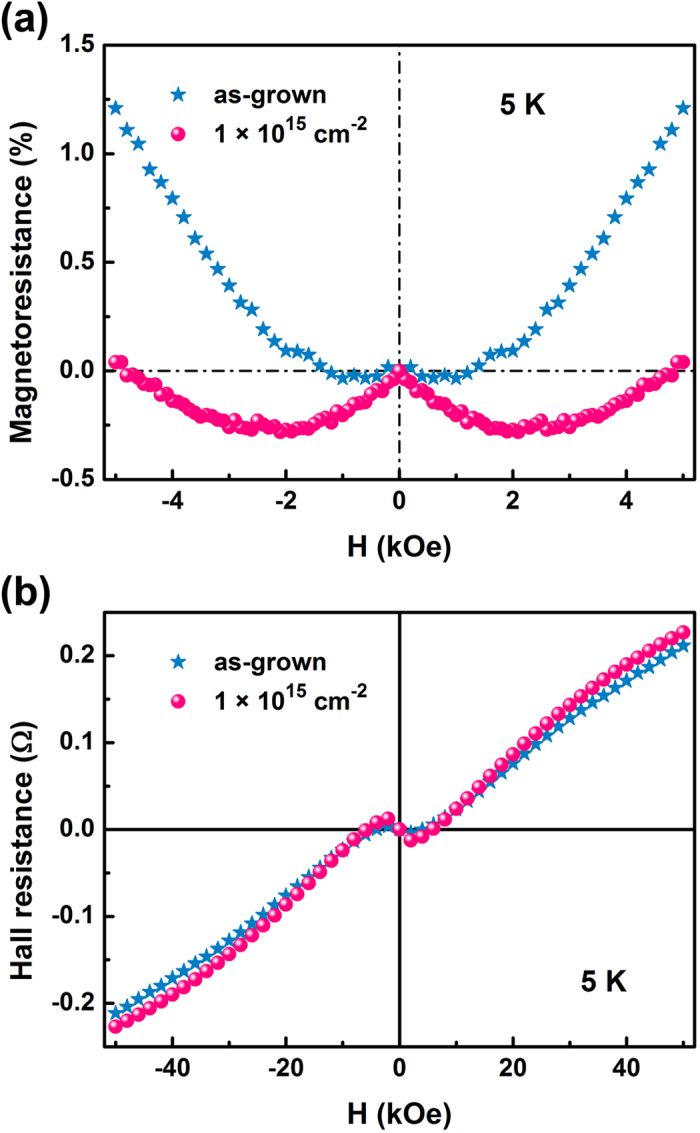
(**a**) The magnetoresistance and (**b**) Hall measurements of vertically aligned graphene before and after implantation. The magnetic field is perpendicular to the substrates and thus parallel to the graphene sheets and the measurement temperature is 5 K.

In as-grown samples, only diamagnetism is observed. After implantation, the magnetism after removing the diamagnetic background is shown in Fig. 6(b). The 1 × 10^15^ cm^−2^ sample has a saturation of 9 × 10^−6^ emu/cm^2^. In the lattice, each implanted Cu ion can bring about 0.97 μ_B_ at room temperature. These localized spins could be induced directly by Cu doping. However, the weak coupling between moments in Cu can not survive at room temperature. The fall of ferromagnetism in the 5 × 10^15^ cm^−2^ sample can not be understood with Cu doping, either. Considering that the implantation also creates defects in graphene, this result may be understood as defect-induced ferromagnetism^40^,^41^,^42^,^43^,^44^. This case implies the feasibility to dope graphene with various elements. In the pristine sample, weak negative magnetoresistance is observed in Fig. 7(a) while the Hall resistivity shows the multiple carriers involved in transport properties according to Fig. 7(b). The two major carriers are the electron with the Hall mobility of 6 × 10^4^ cm^2^/(Vs) and the carrier concentration of about 3 × 10^18^ cm^−3^, and the hole of 1 × 10^4^ cm^2^/(Vs) and 1 × 10^19^ cm^−3^ according to a two-band model^45^,^46^ (Figure S4, Supplementary Information). Contrast to the epitaxial planar graphene, the vertical graphene has less influence from the substrates so it can have better electronic performance. After implantation, magnetoresistance shows the transition from negative to positive when the field is rising at 5 K. The negative magnetoresistance due to weak localization can be understood as the implantation induces impurities and defects as scattering centers. The implantation ameliorates the electron Hall mobility to 9 × 10^4^ cm^2^/(Vs) while the concentration reduces slightly to 2 × 10^18^ cm^−3^. The hole Hall mobility and concentration almost remain unchanged after implantation. It is speculated that the impurity band of Cu is located a little bit below the major negative carrier so Cu doping can shift the Fermi level towards the Dirac cone and thus increase the mobility.

In conclusion, the growth control of vertically aligned graphene on C-face SiC has been investigated. The influence of processing conditions is demonstrated. A temperature higher than 1700 °C is indispensable and the growth will accelerate with temperature increase. The graphene height is basically proportional to the annealing time. Proper temperature gradient and good crystalline quality of SiC substrates can further associate the vaporization of substrates and promote the growth. Pressure does not directly affect the growth while the partial pressure of vapor is crucial as it can interfere with further vaporization. The growth mechanism is proposed in terms of physical vapor transport. The monolayer character is verified by Raman and XAS spectra regardless of the graphene height, which indicates the robustness of the growth method. With the obtained samples, the d^0^ magnetism of 0.97 μ_B_ per Cu at 300 K is revealed in Cu-implanted vertical graphene. The negative magnetoresistance due to weak localization after implantation is observed. Vertical graphene possesses both electron and hole as major carriers, whose Hall mobility is more than 1 × 10^4^ cm^2^/(Vs). In implanted samples, its electron Hall mobility is further improved to 9 × 10^4^ cm^2^/(Vs). The results will be helpful to extend the research area and the potential application of vertically aligned graphene.

## Methods

### Substrates and sample preparation

We used commercially available SiC substrates (Beijing TankeBlue) to grow vertically aligned graphene. Without special notation, the substrates are conductive C-face 4H-SiC of dummy level with dimensions of 10 × 5 × 0.33 mm^3^.

**Figure 8:**
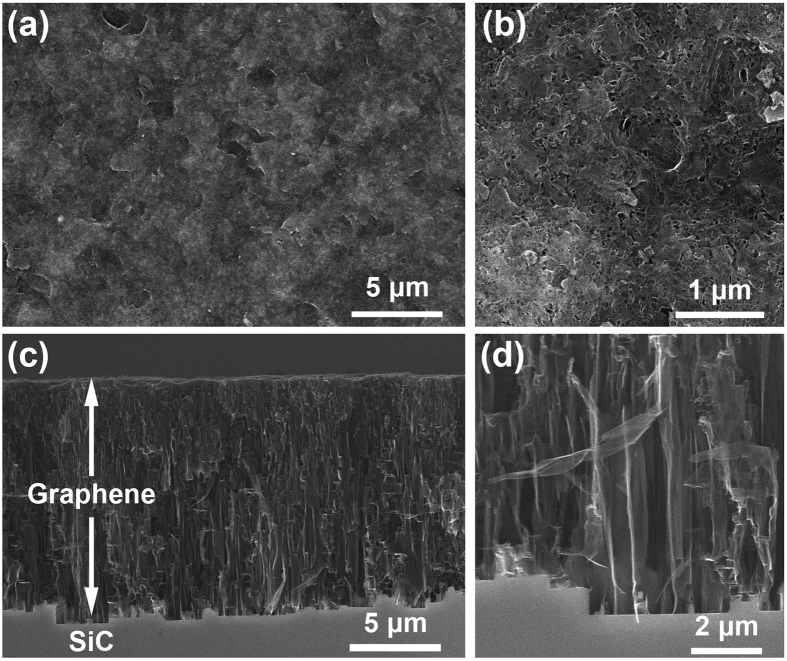
Morphology of vertically aligned graphene imaged in a scanning electron microscope. (**a**,**b**) Top view and (**c,d**) cross-sectional view at different magnifications. The substrate was annealed with a temperature ramping from 1800 °C to 1900 °C within 2 hours under a pressure of about 10^−5^ mbar.

The growth system is a high-temperature annealing furnace (Jipelec, SiC Furnace) with an induction coil and a turbo molecular pump, which can reach temperatures as high as 2000 °C and a vacuum of ~10^−6^ mbar. SiC substrates with C-side facing up are loaded into a graphite susceptor with a lid. The susceptor is placed in the middle of the chamber supported by a graphite tubular mount (Figures S1 and S2, Supplementary Information). The temperature is measured from the bottom of the susceptor by an infrared thermometer. A typical recipe is to anneal the substrates with a temperature ramping from 1800 °C to 1900 °C within 2 hours under a pressure of about 10^−5^ mbar. Figure 8 shows the quintessential micro morphology. Generally, the surface of the samples is not smooth as with non-polished SiC the substrate surface is rough [Fig. 8(a)]. Cavities distributed irregularly across the sample surface are due to the etching of high-temperature vapor [Fig. 8(b)]. The cross-sectional image in Fig. 8(c) indicates that the graphene ribbons are aligned vertically. Representative dimensions of these graphene ribbons are 4 μm in length and 0.5 μm in width [Fig. 8(d)]. These results are similar to observations in previous reports^11^,^30^.

Some vertical graphene samples were implanted with Cu ions after annealing. The incident energy and angle were 190 keV and 7°, respectively. The samples with the Cu fluence varying from 1 × 10^14^ to 1 × 10^16^ cm^−2^ are labeled according to their fluence. Cu distribution is demonstrated in inset of Fig. 6(a). The doping level can be estimated as fluence multiplying distribution. For example, the doping level is about 10^20^ cm^−3^ for the sample with the Cu fluence of 1 × 10^16^ cm^−2^.

### Sample characterization

Scanning electron microscopy (SEM) micrographs were captured using an S-4800 field-emission SEM (Hitachi) operated at an acceleration voltage of 10 kV. Room temperature Raman measurements were carried out at a LabRAM (HORIBA Jobin Yvon) using a 532 nm Nd:YAG laser as excitation source with a resolution of ~0.5 cm^−1^. X-ray absorption spectroscopy (XAS) was performed at beamline 6.3.1 of the Advanced Light Source using the total electron yield mode at room temperature. Rutherford backscattering (RBS) spectrometry with ~1.7 MeV He ions was employed to determine the composition distribution. The magnetic properties were measured by a vibrating sample magnetometer equipped with a commercial superconducting quantum interference device (SQUID-VSM, Quantum Design) with a sensitivity limit of 10^−7^ emu in the field range of 5 kOe at 300 K. The transport measurements were executed using a current of 1 mA under the magnetic field of 50 kOe at 5 K with a Lakeshore system.

## Additional Information

**How to cite this article**: Liu, Y. *et al*. Controllable growth of vertically aligned graphene on C-face SiC. *Sci. Rep.* **6**, 34814; doi: 10.1038/srep34814 (2016).

## Supplementary Material

Supplementary Information
