# Supplementary material for: Controllable growth of vertically aligned graphene on C-face SiC
Source: Sci Rep. 2016 Oct 6;6:34814. doi: 10.1038/srep34814 (PMC5052588; doi:10.1038/srep34814)
Supplement: Supplementary Information [file srep34814-s1.pdf]

Supplementary Information

Controllable growth of vertically aligned graphene on C-face SiC

Yu Liu[1], Lianlian Chen[2], Donovan Hilliard[1,5], Qing-song Huang[3], Fang Liu[1,6], Mao Wang[1,6], Roman Böttger[1], René Hübner[1], Alpha T. N'Diaye[4], Elke Arenholz[4], Viton Heera[1], Wolfgang Skorupa[1], Shengqiang Zhou[1]

[1]Helmholtz-Zentrum Dresden-Rossendorf, Institute of Ion Beam Physics and Materials Research, 01328 Dresden, Germany

[2]Department of Basic Science, Beijing Information Science and Technology University, Beijing 100192, China

[3]School of Chemical Engineering, Sichuan University, Chengdu 610065, China

[4]Advanced Light Source, Lawrence Berkeley National Laboratory, Berkeley, California 94720, USA

[5]School of Physics, Dublin institute of technology, Dublin, Ireland

[6]Technische Universität Dresden, 01062 Dresden, Germany

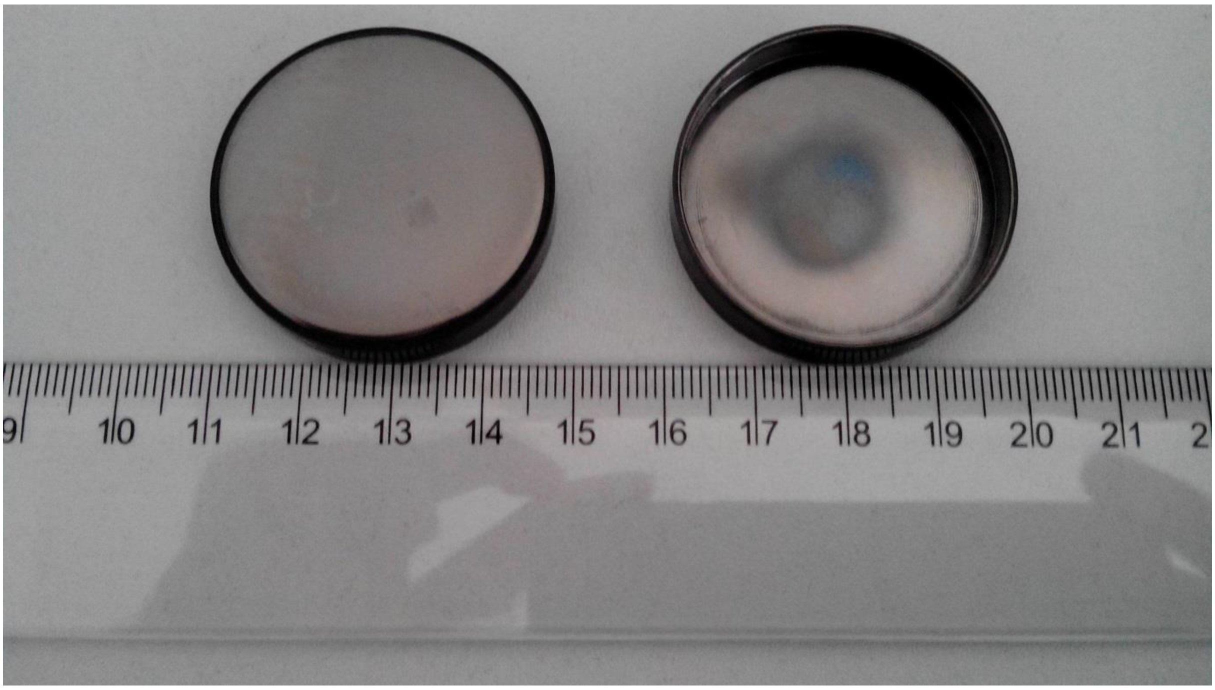

Figure S1. The graphene susceptor and its lid.

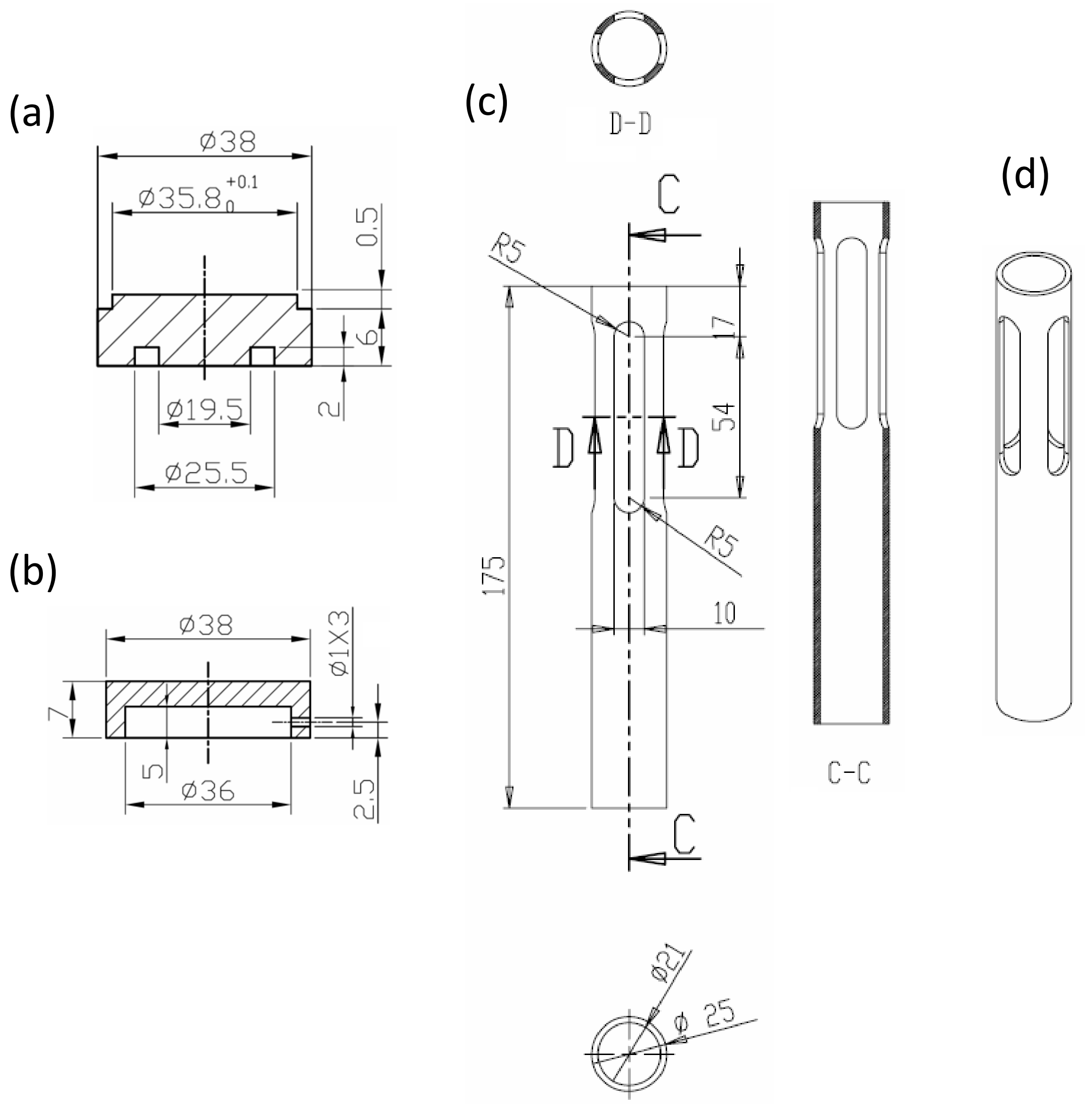

Figure S2. (a) The mechanical drawing of the graphene susceptor with a bottom thickness of 6 mm, (b) the graphene lid, and (c) the graphite tubular mount with a height of 175 mm. (d) Overall view of a graphite tubular mount.

2 inch diameter Silicon Carbide (SiC) Substrate Specification

等级 Grade	工业级 Production Grade	研究级 Research Grade	试片级 Dummy Grade
直径 Diameter	50.8 mm±0.38 mm (2.000″±0.015″)		
厚度 Thickness	330/430 μm±25μm		
晶片方向 Wafer Orientation	On axis : <0001>±0.5° Off axis : 1.0°/3.5°/4.0/8.0 toward <11$\bar{2}$0>±0.5°		
主定位边 Primary Flat	{10-10}±5.0°		
主定位边长度 Primary Flat Length	15.88 mm±1.65 mm (0.625″±0.065″)		
次定位边长度 Secondary Flat Length	8.0 mm±1.65 mm (0.315″±0.065″)		
次定位边方向 Secondary Flat Orientation	Si-face:90° cw. from orientation flat ± 5°/C-face:90° ccw. from orientation flat ± 5°		
边缘 Edge exclusion	1 mm		
总厚度变化/弯曲度/翘曲度 TTV/Bow /Warp	≤25μm /≤25μm /≤25μm		
微管密度 Micropipe Density	≤10 cm^{-2}	≤30 cm^{-2}	≤100 cm^{-2}
半高宽 FWHM	<30 arcsec	<50 arcsec	Na
电阻率 Resistivity	4H-N 0.01Ω·cm ~0.03 Ω·cm	4H-N 0.01Ω·cm ~0.1Ω·cm	4H-N 0.01Ω·cm ~0.1Ω·cm
	6H-N 0.02Ω·cm ~0.1 Ω·cm	6H-N 0.02Ω·cm ~0.2Ω·cm	6H-N 0.02Ω·cm ~0.2Ω·cm
	6H-SI (90%) >1E5 Ω·cm	6H-SI (80%) >1E5 Ω·cm	6H-SI (70%) >1E5 Ω·cm
表面粗糙度 Roughness	Polish Ra≤1 nm	Polish Ra≤1 nm	N/A
	CMP Ra≤0.5 nm	CMP Ra≤0.5 nm	N/A
裂纹(强光灯观测) Cracks by high intensity light	Edge<1mm	Edge1-2mm	Cumulative length≤10mm,single length≤2mm
六方空洞（强光灯观测） Hex Plants by high intensity light	2 allowed ≤100 microns each	5 allowed ≤300 microns each	Cumulative area≤30%
多型(强光灯观测) Polytype Areas by high intensity light	None	Cumulative area≤2 %	Cumulative area≤5%
划痕(强光灯观测) Scratches by high intensity light	None	None	3 scratches to 1×wafer diameter cumulative length
表面污染物（强光灯观测） Contamination by high intensity light	None	None	None
有用面积 Usable area	≥90%	≥80%	≥70%

Figure S3. Specifications of 2-inch diameter SiC substrates from TankeBlue Semiconductor Co. Ltd. FWHM in Line 11 is the full width at half maximum of the rocking curve at (006) for 6H or (004) for 4H in X-ray single crystal diffraction, which is a direct indication of the overall crystalline quality of the wafer. The requirement of a production grade wafer is < 30 arcsec while it is not requested for a dummy grade wafer. Therefore, the substrates from the production grade wafers have better crystalline quality than those with the dummy grade.

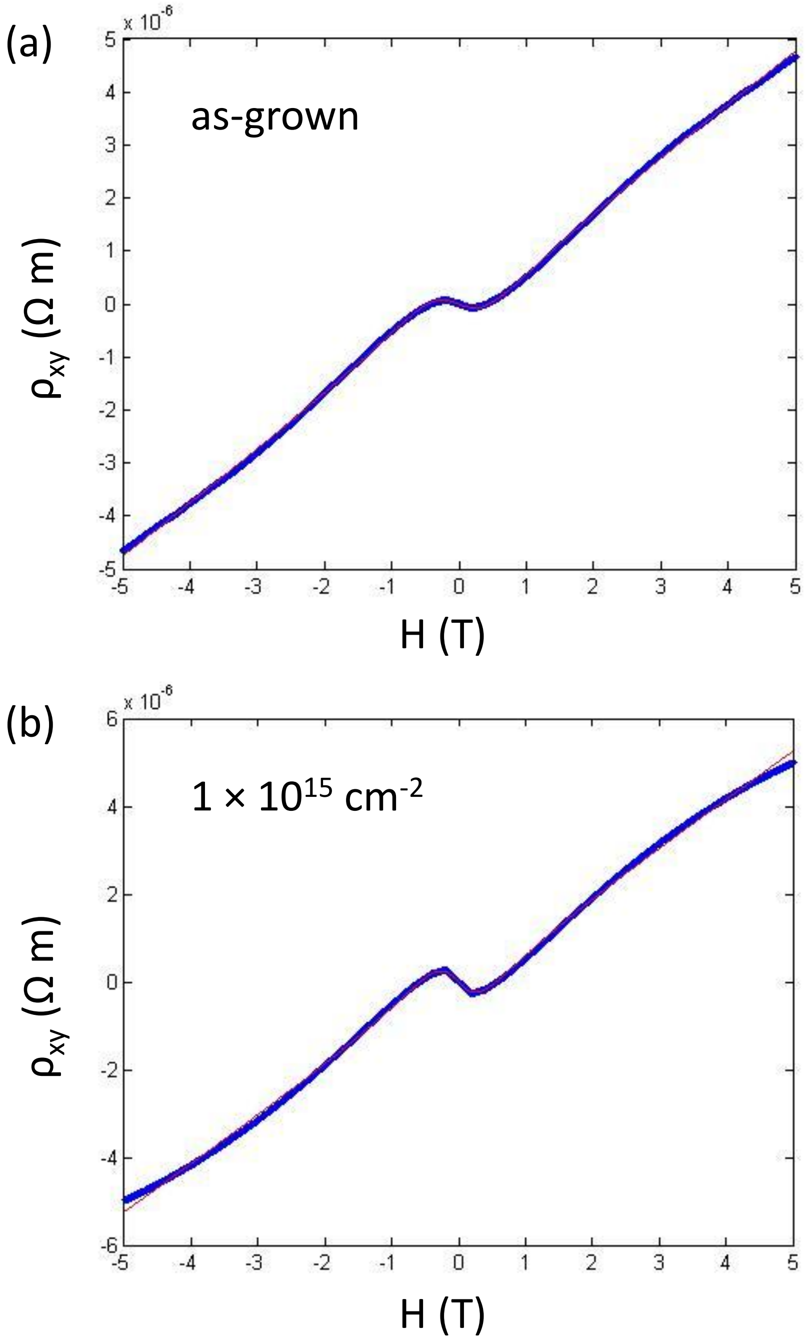

Figure S4. Hall resistivity of as-grown (a) and Cu ion implanted (b) vertically aligned graphene. Blue thick lines are data from measurements and red fine lines are fitting results. The Hall resistivity ρ_{xy} are fitted with a two-band model:

$$\rho_{xy} = \left(\frac{B}{e}\right)\frac{(n_e\mu_e^2 + n_h\mu_h^2) + B^2\mu_e^2\mu_h^2(n_e + n_h)}{(n_e\mu_e + n_h\mu_h)^2 + B^2\mu_e^2\mu_h^2(n_e + n_h)^2},$$

$$\rho_{xx} = (en_e\mu_e + en_h\mu_h)^{-1},$$

where n_e (or n_h) is the carrier concentration of electrons (or holes) and μ_e (or μ_h) is the Hall mobility of electrons (holes).

Table S1. Process parameters of four samples in Raman measurements.

Label	Graphene height	Temperature (°C)	Time (h)	Susceptor thickness	Mount height	C-side facing	Pressure (mbar)
**G140923**	1.3 μm	1600 - 1700	10	6 mm	175 mm	Down	10^{-5}
**G150205-2**	7.7 μm	1800 - 1900	2	10 mm	175 mm	Up	10^{-5}
**G150305-2**	38.5 μm	1700 - 1800	10	10 mm	175 mm	Up	10^{-5}
**G150310**	246.4 μm	1800 - 1900	40	10 mm	175 mm	Up	10^{-5}
